# What Changes Occur in the Teeth with the Advancement of Age

**Published:** 1892-11

**Authors:** V. A. Latham


### COMMUNICATIONS.

### What Changes Occur in the Teeth with the Advancement of Age.

By V. A. Latham, D. D. S.

To understand how age alters the teeth it is necessary to understand how they are nourished. Besides the pulp, peridental membrane and the main arteries we find a decided lymphatic system, or in other words, the ever-acting powers of osmosis are being carried on. This circulation is maintained in the fibrillæ of the dentinal tubuli, and life is thereby sustained in the dentine. As the tubuli anastomose with the canaliculi of the cementum at the periphery of the dentine, and the circulation is continuous between the two tissues, we depend on this circulation for the maintenance of life, sufficient for the toleration of the tooth by the living tissues about it, after the removal of the life source of the dentine, the pulp. We expect it to preserve not only the life of the cementum intact, but also to maintain some vitality in the dentine in contact with it.

Teeth at eruption are not so dense in structure, so rich in inorganic elements as at maturity. Again, this density usually increases with age and active employment, so that the dentine of old age and the dentine of adolescence are very different in quality. The former is nearly devoid of protoplasm, and the fibrils become calcified to some extent, and often the pulp itself; while the latter, though morphologically perfect is very incomplete chemically, and possesses a large quantity of mere protoplasm, which will need to be calcified before the dental tissues will reach their mature texture. Therefore, if this calcification can take place after the tooth is erupted and morphologically complete, we must believe from analogy that the polarity can be reversed and decalcification be possible under incitement of pathological conditions. Even physiological change of the circulating fluids, such for example as occurs in pregnancy, induced perhaps by lime starvation, may cause molecular change, for we have every reason to believe that lime is taken from the teeth and the bones for the complete osseous system of the fœtus and it is returned after the function is completed. Lactation also causes a disturbance and a draining of the system, though of a somewhat different kind, if the required lactic elements are not sufficient to meet the demand.

We are taught by physiologists that there is a constant splitting up of the molecules of which the body is composed, giving rise to different tissues and combinations in different parts of the body. Therefore, every deviation from the normal condition, no matter how slight, will interfere in the proper makeup and action of the organs, the teeth being peculiarly liable to suffer from the general malnutrition, inducing a chronic starvation, and by lowering their vitality and robbing them of much of this reserve store of materials, render them more liable to caries, waste of the inorganic elements of dentine and probably of enamel without compensation for the loss, thus carious attacks are easy. Again, there may be increase of density as transpires with age, by a more rapid construction than waste. If some of the teeth are lost and the patient requires much soft food, as the remaining teeth are thus prevented from getting a certain amount of antagonistic pressure which they require to retain them in the jaws, these teeth become loosened and elongated, which is a condition often seen in elderly patients.

If we examine the teeth of childhood and youth, whether temporary or permanent, we find them possessed of certain characteristics belonging quite uniformly to this period of life. The dentine and enamel are comparatively soft and unconsolidated and of exalted nervous organization; a preponderance of animal matter throughout the tooth, with lime-salts lacking both in quantity and arrangement, all yielding readily to decay in localities favoring the action of external destroying agents. The younger the tooth or the more imperfectly formed and consolidated, the more unfortunate to that tooth, then, would be the death of its pulp.

When the pulp has effected its work in the organization of a perfect tooth of the same use and value as in the earlier periods of formation and nourishment, the pulp seemingly rests from its labors for a time and only takes on activity again when later in life it becomes an organ of destruction, for it is often seen that teeth with living pulps which at maturity and on to the period of systemic decline were dense, strong and decay-resisting, in old age speedily become fragile or soft and readily yield to decay. This seems, therefore, to point out that we must endeavor by all means to save the pulp in youth and adult life, but in more matured teeth its preservation is not of such vast importance to the tooth, and that in the decline of life the pulp may become the means of undermining the solid and resisting combinations found at maturity and thus exposing formed teeth to decay.

The tooth pulp differs in size and character according to age; the odontoblasts vary very much in form; in young pulps before the formation of dentine, they are roundish, or pyramidal; during their greatest functional activity the end toward the dentine is square and tapering, whilst in old age they become almost unseen and assume a somewhat ovoid shape. The pulp diminishes in size by progressive calcification and the pulp-cavity lessens. The pulps may also, in advancing age atrophy; the connective tissue is more abundant whilst the cellular elements diminish. The capillary system is obliterated and a fatty degeneration of the nerves takes place and the tooth is loosened in its socket.

Secondary dentine occurs from many causes, but it is also found as a pathological condition in aged teeth in which the pulp cavity is much contracted in size, and also very frequently found as a protective layer when a pulp is threatened by the approach of caries by the thinning of the walls of the pulp cavity through excessive wear. The dentinal tubules are also obliterated and even calcified, as there is the resemblance in the appearance of the translucent zone to that of the roots of old teeth which are supposed to be more rich in lime salts than ordinary healthy dentine. In young teeth the dentinal fibres are present and easily stain with carmine in the finest peripheric ramifications of the tubules, but in those that are older atrophy of the fibres appears to be concurrent with obliteration of the canaliculi. The younger fibres possess a remarkable degree of extensibility, so that the dentinal cells may be separated to a considerable distance from the dentine without rupture of the processes, which then appear like harp strings stretched across the interval. In young fresh teeth of a calf, rounded and stellate cells may frequently be seen in the larger interglobular spaces, with processes which extend into the dentinal canals opening into them. The teeth seem, as years advance, to become coated with tartar to a much greater extent than even in adult life, and it is possibly, owing to change in the saliva and alimentary canal through dyspepsia, gout, rheumatism, etc., in which diseases tartar is often found in abundance; through it the gums are injured and the teeth irritated, loosened and elongated.

After the enamel is formed and calcified few changes, apparently, occur in it except as decay, though it hás been seen with polariscopic light, the developed enamel exhibits strongly negative double refraction, whilst young enamel presents positive double refraction, showing, evidently, a change in the substance. The adult enamel becomes positive, however, if exposed to a temperature of 800°. C. Hoppe-Seyler gives in Virchow's Archives (Bdv Bd xxiv Zahnschmelz) a chemical analysis of the difference of composition in young and old ènamel. In that of a newly-born infant the organic compounds are about 15-59, whilst the enamel of adults contains only 1-3 per cent. organic constituents, but on the other hand a large quantity of phosphate of lime. A remarkable feature I think in the analysis is the presence of a small proportion of fluorine. As yet little seems to be known of the changes age produces in the teeth and it will only be accomplished by making comparative chemical analyses of the various constituents of young and old teeth and also microscopical specimens to determine the various properties as to structure, appearance, etc., and any other conditions likely to be found which may be caused through increased or diminished circulation, as the arteries in advancing years become somewhat changed and may thus interfere with the nourishment of the mouth, teeth and associated parts to some extent.

